# Assessing *S. mansoni* prevalence in *Biomphalaria* snails in the Gombe ecosystem of western Tanzania: the importance of DNA sequence data for clarifying species identification

**DOI:** 10.1186/s13071-017-2525-6

**Published:** 2017-11-23

**Authors:** Jared S. Bakuza, Robert Gillespie, Gamba Nkwengulila, Aileen Adam, Elizabeth Kilbride, Barbara K. Mable

**Affiliations:** 1 Institute of Biodiversity, Animal Health and Comparative Medicine, College of Medical, Veterinary and Life Sciences, University of Glasgow, Glasgow, UK; 2 Department of Biological Sciences, Dar es Salaam University College of Education, Dar es Salaam, Tanzania; 3 Department of Zoology and Wildlife Conservation, University of Dar es Salaam, Dar es Salaam, Tanzania

**Keywords:** PCR diagnostics, Cercarial shedding, Prevalence, *Schistosoma mansoni*, *Biomphalaria pfeifferi*

## Abstract

**Background:**

Snails are essential for the transmission and maintenance of schistosomiasis in endemic areas, as they serve as intermediate hosts for schistosome parasites. A clear understanding of the snail species present, their local distribution and infection status is therefore a prerequisite for effective control of schistosomiasis. The purpose of this study was to establish the infection status and distribution of *Schistosoma mansoni* in snails in the Gombe area along the shores of Lake Tanganyika in western Tanzania, using both detection of cercarial shedding and molecular approaches.

**Methods:**

Snails were collected from streams located close to human settlements in Gombe National Park, as well as from nearby villages (Kiziba, Mtanga, Mwamgongo and Bugamba) and the largest town in the region (Kigoma). Snails were individually exposed to light to induce shedding of schistosome larvae, which were examined using a compound light microscope. Additionally, the internal transcribed spacer (ITS) region of the ribosomal RNA gene cluster was simultaneously amplified in both snails and their trematodes using a single polymerase chain reaction (PCR) and sequenced to confirm species identification.

**Results:**

Snails morphologically identified as *Biomphalaria pfeifferi* were present in all streams except at Mtanga but their distribution was patchy in both time and space. Sequencing of PCR products indicated that not all snails were *B. pfeifferi*. None of the snails from Gombe or Bugamba shed schistosome larvae, while larvae were shed at all other sites. Overall, an infection prevalence of only 12% was observed in snails based on cercarial shedding. While 47% of the snails were PCR-positive for the 500 bp ITS fragment, which was predicted to indicate infection with *S. mansoni*, sequence data demonstrated that these bands are not species-specific and can be amplified from other trematode infections. In addition, a 1000 bp band was amplified in 14% of samples, which was identified as a trematode in the family Derogenidae.

**Conclusions:**

The results support the previous assumption that *B. pfeifferi* snails may be involved in transmitting schistosomiasis in the area but suggest that the community structure of both snails and trematodes may be more complicated than previously thought. This emphasises the importance of confirming species identifications using sequencing, rather than relying only on PCR-based diagnostics or cercarial shedding.

**Electronic supplementary material:**

The online version of this article (10.1186/s13071-017-2525-6) contains supplementary material, which is available to authorized users.

## Background

Studies on the transmission of intestinal schistosomiasis caused by *S. mansoni* (family Schistosomatidae) in humans have shown that the disease is acquired in areas where people come in contact with a water body containing infected snails [1]. The role of snail intermediate hosts in the transmission and maintenance of schistosomiasis is thus very important. Hence, knowledge of snail distribution and habitat preference is a crucial tool for understanding the epidemiology and control of the disease [2].

Different species of *Schistosoma* have a particular set of snail species that act as competent intermediate hosts [3] but this is complicated by the fact that most of the snail hosts belong to species complexes that have not always been fully disentangled [3, 4]. In Tanzania, for example, urogenital schistosomiasis (caused by *S. haemotobium*) is transmitted by four snail species belonging to the genus *Bulinus*. Of these, *Bu. nasutus* and *Bu. globosus* are widely distributed in the country both on mainland Tanzania and on the islands of Zanzibar (Unguja and Pemba), whereas *Bu. africanus* and *Bu. truncatus* are only found on the mainland [5]. Intestinal schistosomiasis (caused by *S. mansoni*) is transmitted by planorbid snails of the genus *Biomphalaria*. Of these, *B. pfeifferi* is thought to occur in most parts of the country except on the coastal belt and the islands of Zanzibar [6, 7], whereas *B. sudanica* is found in the northern part of the country and *B. choanomphala* is confined to the shores of Lake Victoria [5, 6]. However, clear identities and distribution of snail species and their role as vectors of schistosomiasis are poorly known, particularly in the western part of Tanzania [7]. For example, Lake Tanganyika has been regarded as possibly being schistosomiasis-free because most of its shores are rocky and constantly pounded by heavy waves, which might be detrimental to snail survival [8]. However, the lake is fed by multiple mountain streams, which could provide suitable snail habitats. Snails identified as *Biomphalaria* have been found in Gombe National Park close to the shores of Lake Tanganyika and *S. mansoni* infections have been diagnosed in non-human primates in the areas [9]. However, which snail species were present and capable of transmitting the parasites within the park and in the surrounding human-inhabited areas was not established.

Furthermore, a recent study [10] found unexpectedly high prevalence of *S. mansoni* in humans in Gombe and adjacent areas, with Kato-Katz detection of eggs from faeces in up to 45% of adults and 90% of children sampled in the villages adjacent to the park. However, there was extensive variation among villages in the number of positive individuals. It is possible that physical barriers to snail dispersal in the area caused by mountains and valleys may have caused low or no migration of snails between villages, which could result in variation between sites in the presence or absence of snails and their schistosome infection status. The highest prevalence of *S. mansoni* was in the village (Mwamgongo) closest to the Gombe National Park, which could increase the risk of cross-transmission between humans and non-human primates if animals and humans share water sources containing competent snail hosts. This hypothesis is difficult to assess without detailed knowledge about the distribution of snail species in the area and their infection status with schistosomes.

Diagnosis of schistosomes in their snail intermediate hosts has traditionally relied on exploiting the propensity of snails to shed cercaria when exposed to light, using microscopy identification of schistosome cercariae [11]. However, a limitation of this approach is that it can only identify patent infections [12]. It is also difficult to distinguish between other closely related trematodes infecting vertebrate hosts, such as the rodent-associated *S. rodhaini*, which is also in the *S. mansoni* group and can hybridise with *S. mansoni* [13, 14]. Other approaches have thus been developed for diagnosing infections in humans and snails [1], with some exploiting antibodies produced by infected individuals [15] or molecular techniques targeting repeat regions of DNA [12, 16, 17]. Molecular-based identification using the polymerase chain reaction (PCR) can be more sensitive at detecting low levels of infection, but the relatively higher cost and inefficient use in the field means that microscopy has instead been promoted and is still preferable under some situations [18]. However, an advantage of PCR over microscopy-based techniques is the ability to diagnose multiple infections with different parasites [19], particularly when combined with sequencing to confirm species identification [20]. A particularly common approach is to base such diagnostics on amplification of the internal transcribed spacer regions (ITS) of the ribosomal DNA array because of extensive length polymorphism that can be used to distinguish certain parasite species [21, 22]. This can be combined with restriction enzyme digestion to enable detection of variants even within species in some cases [22], which can provide a rapid and cheaper means of screening large number of individuals than sequencing. However, unless at least some representative variants are sequenced, there is a risk that new species, genetic variants within species, or closely related species (such as *S. rodhaini*) will be mis-assigned based only on fragment size. In principle an additional advantage of using the ITS region is that there is potential to simultaneously amplify the host or vector and the parasites using a single PCR because universal conserved primers can be designed in the flanking genes (18S and 5.8S) [23]. However, this strategy has not previously been applied to trematodes. Such an approach would be beneficial because amplification of the host or vector DNA can act as an internal control of DNA quality to interpret presence or absence of parasites and it is cheaper and easier for field-based surveys than using specific primers to target each species separately.

The purpose of this study was to identify the species of snail vectors and parasites present in the Gombe National Park ecosystem along Lake Tanganyika. PCR-based screening using general ITS1 primers that amplify DNA from both snail hosts and trematodes was initially compared with microscopy-based cercarial shedding, to test their relative sensitivity and specificity. Bands predicted to correspond to particular species of snails and parasites were then sequenced, to determine whether there was more variation present than would have been predicted based only on diagnostic PCR. The ultimate aim was to make recommendations for surveillance programmes to monitor transmission dynamics of schistosome prevalence in snails, humans and non-human primates in the Gombe area. Results are thus discussed in relation to *S. mansoni* infection status of humans and baboons sampled from the same geographical region [10].

## Methods

### Snail collection and identification

**Fig. 1:**
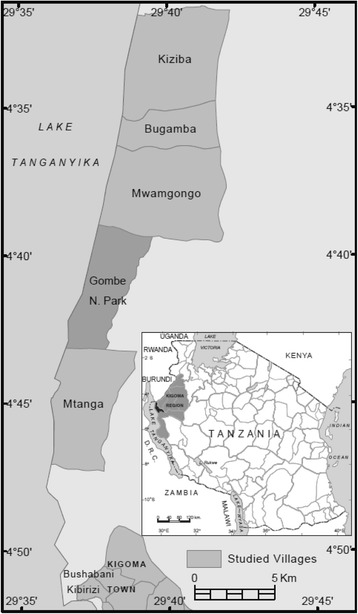
The location of the study area (Gombe ecosystem) in the Kigoma region of western Tanzania (inset). The sampled streams from bottom to top are: Nyakageni in the Kibirizi area along Kigoma Bay in Kigoma town, Mtanga in Mtanga village, Kakombe and Mkenke in Gombe National Park; Ngonya in Mwamgongo, Misonga in Bugamba, and Bumba and an unnamed stream in Kiziba

The study was conducted in the Gombe ecosystem along the shores of Lake Tanganyika in western Tanzania (Fig. 1). Sampled streams were strategically chosen to be in close proximity to human activities. Snails were sampled from streams at Gombe National Park (Kakombe and Mkenke) and four of the surrounding villages: Kiziba (Bumba and unnamed stream), Mtanga (Mtanga), Mwamgongo (Ngonya) and Bugamba (Misonga) (Fig. 1, Additional file 1: Table S1). In addition, one stream (Nyakageni River) within the largest urban area in the Kigoma Municipality (Kigoma Town) was included. Twelve sampling points were selected along each stream by pacing out the length of the river, with each point being located at least 200 m apart. The same sites were visited towards the end of the dry season (September) and in the wet season (October-April). Snails were systematically searched for at each point for a period of 15 min and sampled using a scoop (30 by 30 cm) covered with a 2 × 2 mm size mesh [24]. Individual snails were placed in separate plastic vials (120 ml) and the containers wrapped with aluminum foil to keep light away and prevent the snails from shedding cercaria larvae before reaching the laboratory. Samples were analysed microscopically on the day of collection in a baboon research room at Gombe Stream Research Centre or at Mwamgongo Health Centre. Snails were identified morphologically as belonging to the genus *Biomphalaria* based on their blackish sinistral (left) coiled shell, following published identification guidelines [3] and descriptions from previous reports on snail sightings in the region [9, 25].

### Shedding of parasite larvae (cercariae) from snails

Snails were individually washed with distilled water and transferred into a well of 12- or 24-well plates containing filtered stream water. The plates were then exposed to artificial light (60 W) to enable the snails to shed cercariae [11]. After a period of 6 to 12 h, each snail was observed under a dissecting microscope to determine if they were shedding schistosome larvae. Larval cercariae shed from the snails were morphologically identified to the genus level [3, 26]. Each snail was then dissected and its tissue preserved in a 1:3 volume ratio of tissue to RNA-later solution at room temperature until required for DNA extraction [27].

### DNA extraction and PCR amplification of snail tissues

DNA extractions and PCR amplifications from snails were conducted at the University of Glasgow. Snail tissues were chopped into small pieces and DNA was extracted from up to 100 mg using DNeasy® Blood and Tissue Kits (Qiagen Inc., Manchester, UK). Polymerase chain reactions targeting the internal transcribed spacer region (ITS1) of the rDNA array were performed, using the primers ETTS2 (5′-TAA CAA GGT TTC CGT AGG TGA A-3′) and ETTS17 (5′-CGA GCC GGA TGA TCC ACC GC-3′). Although these primers were designed to amplify multiple species of snails [20, 22], they also amplified trematode DNA [10], enabling a single PCR to confirm the identity of vectors (or hosts) and parasites. PCR was carried out in 20 μl reactions containing 500 nM of each primer, 1× Taq Reaction buffer, 2.5 mM magnesium chloride, 0.05 U Taq Polymerase (Invitrogen, Inc., Paisley, UK), 0.2 mM dNTPs and 2 μl DNA. Reaction volumes were brought to 20 μl with water. The PCR profile consisted of an initial denaturing step at 95 °C for 3 min, followed by 34 cycles of denaturing at 95 °C for 45 s, annealing at 55 °C for 60 s, and extension at 72 °C for 60 s. The cycle was completed with a final extension at 72 °C for 5 min. For each set of reactions, a negative PCR control (using distilled water), and negative extraction controls (using just the extraction kit reagents) were run to check for contamination in the reagents used. A positive control using *S. mansoni* DNA extracted from faecal samples from known infected baboons in Gombe National Park was also run, to confirm the size band expected for the parasite.

To ensure that lack of amplification of parasites was not due to poor DNA quality, two approaches were used. First, only samples for which the snail band amplified cleanly were used for assessment of prevalence; in cases where this was not true, the PCR was repeated using new DNA extractions or by varying the concentration of DNA added to the PCR. Secondly, the DNA quality and quantity (in ng/μl) was assessed using a Nanodrop® ND-1000 UV-Vis spectrophotometer and Nanodrop ND-1000 (V3.3.0) software. DNA quality was assessed based on the ratio between the absorbance values at 260 nm and 280 nm (260/280); for samples that deviated from a ratio of 1.8–2.0, the DNA extractions were repeated on remaining tissue that had been preserved. Any sample sets showing evidence of contamination in the negative extraction controls were also repeated.

The presence of trematodes was assessed based on an amplification of a 500 bp product (see [10]); species identification was confirmed by sequencing for a subset of samples. Any other size bands that amplified were also sequenced. A target of three infected and three uninfected snails from each population were selected for sequencing, to confirm species identity of both vectors and parasites. Prior to sequencing, amplified bands were excised from the gel and purified using Qiagen Gel extraction kits, using the manufacturer’s protocol (Qiagen, Inc., Manchester, UK). Samples were sequenced at the GenePool (University of Edinburgh) using cycle sequencing with Big Dye and an ABI 3730 sequencer. Chromatographs were visualised and base-calling errors corrected using Sequencher version 4.5 (Gene Codes, Inc. Ann Arbor). Sequence identity of each amplified band was confirmed using mega BLAST (as implemented on the National Centre for Biotchnology Information, NCBI web portal) to identify the most similar sequences deposited to the GenBank nucleotide database.

## Results

### Snail distribution and shedding of parasite larvae (cercaria)

Snails were found in all streams except in Mtanga village, but their distribution was very patchy both in space and time (Additional file 1: Table S1). The highest numbers were found at Mwamgongo (*n* = 95 in September and *n* = 13 in January), with seven of the 12 sampled points showing presence in September compared with only two in January. At the other sites, snails were predominantly found at one sampling time and at only one location along the streams. All collected snails were morphologically identified as *B. pfeifferi*.

**Fig. 2:**
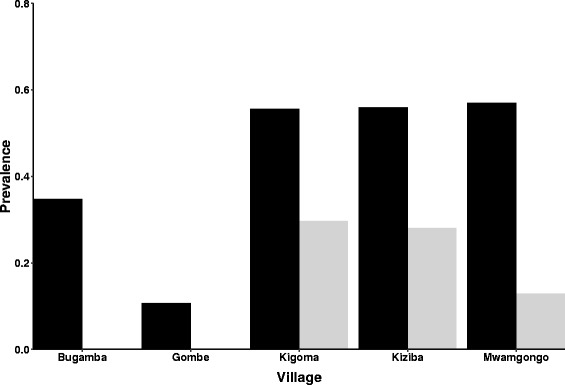
Variation of *S. mansoni* infection in snails sampled from Gombe National Park and surrounding villages. Infection is based on percentage of snails sampled that shed larvae (light bars) or showed presence of a 500 bp amplification product in PCR (dark bars). Note that *S. mansoni* was confirmed based on morphology for shed larvae and its presence was confirmed through PCR in all populations except Kigoma, where the 500 bp band was found to amplify a different species of trematode. Since not all products were sequenced, the presence of *S. mansoni* thus could be overestimated based only on PCR

Morphological examination of the cercaria larvae shed by snails revealed they were elongate and actively swimming and had a forked tail (furcocercous; tail stem longer than furcae) and a tapering head. Across sites, 12.3% of snails shed larvae, but there was extensive variation across streams (Table 1, Fig. 2). Strikingly, there was also a difference between sampling times at some sites: at Mwamgongo only 1/94 snails collected in September shed larvae, in contrast to 13/13 sampled from the same stream positions in January. Based on microscopy-detected cercarial shedding, no infected snails were found from Gombe or Bugamba and the levels of shedding were generally low in the other streams, including 8/30 individuals from Kigoma and 7/29 at unnamed stream in Kiziba village (Additional file 2: Table S2).

**Table 1:** The numbers of snails sampled from the Gombe ecosystem and analysed for trematode infections by cercarial shedding and PCR. Infection prevalence is expressed as percent of snails screened; also indicated is the prevalence of another trematode (family Derogenidae), with a 1000 bp PCR product

Village^a^	No. of snails sampled	Prevalence *S. mansoni* (shedding)^b^	No. of snails screened (PCR)^c^	Prevalence (500 bp band)^c^	Prevalence (1000 bp band)
Bugamba	23	0	23	34.8	26.1
Gombe	46	0	32	0.08	0
Kigoma	30	26.7	30	55.6	0
Kiziba	28	24.1	28	54.2	0
Mwamgongo	108	11.4	106	58.1	22.6
Total/average	235	12.4	219	46.9	13.8

^a^ Mtanga village has been excluded from this table, as no snails were found

^b^ All collected snails were tested for cercarial shedding and all shed cercaria confirmed to be *S*. *mansoni* based on morphology

^c^ Species confirmation through sequencing was made for 8 individuals from Mwamgongo village, one individual each from Kiziba and Bugamba and two individuals from Gombe National Park. For Kigoma municipality, all three products sequenced were from an unknown trematode rather than *S*. *mansoni*

Percentage prevalence based on PCR excludes individuals that did not show clear amplification of the snail band

### Molecular identification of snails and schistosomes

**Fig. 3:**
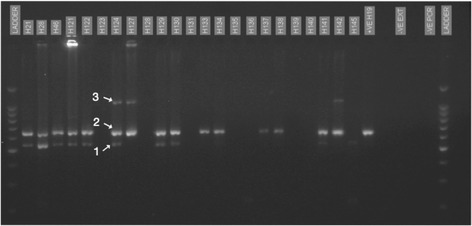
Gel electrophoresis of PCR products of snail DNA with multiple bands. In Lane 8 (H124), the bands for the species detected are indicated by numbers: 1, size predicted for *S. mansoni* (500 bp); 2, size predicted for snails (600 bp); 3, amplification products of an unknown trematode (1000 bp). Also indicated are a positive control (+ve H19), a negative extraction control (−VE EXT) and a negative PCR control (−VE PCR**)**

Out of the 235 sampled snails, PCR products were screened from 219 individuals (Table 1). From these, three size bands were consistently produced in PCR amplifications (Fig. 3): the 500 bp band predicted to correspond to *S. mansoni*, the 600 bp band predicted to correspond to the snail host, and an unknown 1000 bp band. If necessary, PCR for individual samples was repeated 2–6 times to ensure consistency of amplification of the snail band; 39 individuals had strong amplifications on the first attempt and so were not repeated. A total of 27 samples showed consistent lack of amplification of the snail band but three of these showed positive amplification of the 500 bp band; the 24 samples that were negative for all bands were excluded from calculation of prevalence of the parasites. Clearly resolved sequences were obtained for: (i) the 600 bp band from 10 uninfected and 10 infected snails; (ii) the 500 bp band from 14 individuals; and (iii) the 1000 bp band from 4 individuals. Sequencing suggested that while species identification could usually be predicted based on the size fragment, there were some exceptions for both snails and parasites. Due to the extensive length variation, not all sequences could be aligned to one another but we have included an alignment to the closest matching sequences in GenBank, for each of the sequence types found (Additional file 3).

**Fig. 4:**
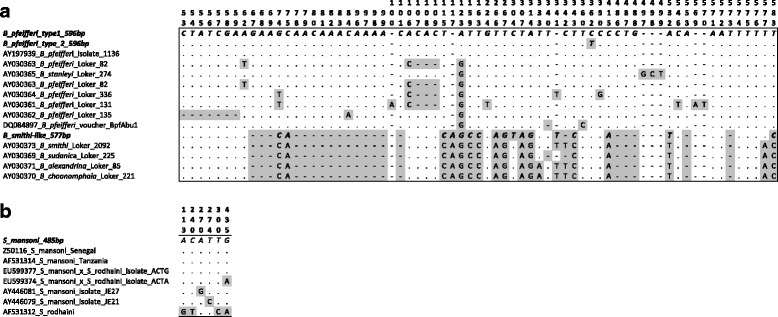
Alignment of sequences of PCR products amplified from the Gombe region to references available in GenBank for snails (**a**) and *S. mansoni*-like (**b**) sequences. Polymorphic positions are indicated (highlighted in grey), numbered from the start of the Gombe sequences (indicated in bold and italics) in each case. The size of the Gombe products are shown in the sequence name. Reference sequences are labelled according to GenBank accession numbers, along with species names and isolates if specified. Note that the *B. smithi*-like sequence differed from the *B. pfeifferi* sequences in indels (indicated by a dash), as well as nucleotide substitutions. There were also some indels in GenBank sequences designated as *B. pfeifferi* and *B. stanleyi*

For the 600 bp snail band, *B. pfeifferi* was confirmed using BLAST for 17 of the individuals sequenced. There was some variation among named *B. pfeifferi* sequences in GenBank, mostly in repeated regions and *B. stanleyi* was within the range of *B. pfeifferi* variation (Fig. 4a). Among the Gombe region samples, there were two different sequence variants, with a single nucleotide fixed difference between them. One type was found at most sites (type 1; GenBank: MG387092) (Fig. 4a, Additional file 2: Table S2 and Additional file 3), whereas the second was found only in the three samples sequenced from Bugamba (type 2; GenBank: MG387093). For the remaining three samples, a different sequence (labelled *B. smithi*-like; GenBank: MH387094) (Fig. 4a) was found for two individuals from Kigoma and one from Gombe (all collected during the wet season) that showed only 93% similarity to the *B. pfeifferi* sequences (i.e. it differed at 45/643 bp). The closest matches in Genbank (99% similarity in each case) were for *B. smithi* (AY030373; 576/578 bp), *B. sudanica* (AY030369; 574/577 bp), *B. alexandrina* (AY030372; 575/578 bp) and *B. choanomphala* (AY030370; 575/578 bp) [4]. However, these were all more similar to one another than to the *B. pfeifferi* sequences (Fig. 4a).

The 500 bp trematode band was amplified in snails from all regions sampled and indicated a higher prevalence of infection than that based on cercarial shedding (Table 1, Fig. 3). After excluding samples that showed lack of amplification of the snail band in repeated amplifications, which might suggest poor quality DNA, 46.9% of samples were PCR positive; all except two snails that shed larvae were also PCR positive. The lowest prevalence of the 500 bp band was found at Gombe (2/32 individuals), where no individuals shed larvae. The most striking difference between the two techniques were at: (i) Bugamba, where a relatively high percent of samples were PCR-positive for the target band (34.8%) but no snails shed cercaria; and (ii) Mwamgongo, where all snails were both PCR- and cercarial shedding-positive in January but 52.4% were also PCR-positive in September, when only a single snail was found to be shedding larvae. Much higher prevalence based on PCR was also found at Kigoma (55.6%) and Kiziba (54.2%) than suggested by cercarial shedding (26.7% and 24.1%, respectively). The 500 bp band was amplified from both *B. pfeifferi* genotypes but not in snails identified as *B. smithi*, which also did not shed cercariae.

Sequences were obtained from 16 individual snails for the 500 bp band. For 13 of these, identical sequences (GenBank: MG387089) were obtained that showed 100% similarity to *S. mansoni* sequences available in GenBank over the 485 bp of sequence obtained (Fig. 4b). These included African (Z50116 [28], AF531341 [29]) and Brazilian isolates (JG289742 [30]), as well as hybrids between *S. mansoni* and *S. rodhaini* (EU599377 [31]). The next most similar sequences were: (i) isolates of *S. mansoni* from Kenya (AY446081 [32]) and Brazil (AY446079 [32]), each showing 99% similarity (468/469 bp); and (ii) *S. rodhaini* (AF531312 [29]), which was also 99% similar but showed more mismatches (465/469 bp) (Fig. 4b).

For three individuals sampled from Kigoma, although the band size was slightly larger but similar to that expected for *S. mansoni* (510 bp of sequence), an unknown trematode was amplified instead (GenBank: MG387090). The sequence was not alignable to the *S. mansoni* sequence and the next closest sequence was the trematode *Macroderoides spiniferus* (EU850403 [33]), which only showed 79% similarity over 78% of the sequence.

The 1000 bp band (Fig. 3) was present only at Bugamba (26.1% of snails screened) and Mwangongo (22.6%). Overall, 14/26 individuals that showed amplification of this band also tested PCR positive for the *S. mansoni* band, but none were observed to shed larvae. The band was sequenced for four snails from Mwamgongo; all were confirmed to be 100% identical to one another (GenBank: MG387091) but there were no similar sequences in GenBank. The closest matches were to trematode parasites with fish final hosts (family Derogenidae) from Japan (e.g. 72% identity over 89% of the sequence for *Genarchopsis gigi*; AB703652 [34]) but the low level of sequence overlap suggests low confidence.

## Discussion

This study has confirmed the presence of schistosome-infected snails in areas along the shores of Lake Tanganyika. Most of the snails were identified as *B. pfeifferi*, corroborating previous reports that the intermediate host for *S. mansoni* [35] occurs in the area [5, 6]. However, our study is the first to identify other species of snails and the different trematodes they carry using DNA sequencing. As consistently concluded by other studies [1], molecular analyses were more sensitive than cercarial shedding in detecting schistosome infections in snails but revealed some cautions about relying only on PCR-based diagnostics for parasite prevalence. Sequencing revealed that not all bands of the target size were *S. mansoni* and not all snails were B*. pfeifferi.* So, prevalence and infection risks could be overestimated if relying only on size fragment-based species identification.

### Molecular identification of snails

Most sequenced snail samples were *B. pfeifferi* but some belonged to a sister clade resolved in recent phylogenetic analyses [4, 36]. All of the species in this clade are thought to be susceptible to *S. mansoni* and are able to transmit the parasite [4, 37]. While the identity of the species was uncertain based on BLAST, any of the four candidates would represent a range extension to published distributions. *Biomphalaria smithi* has been reported in the nearby Democratic Republic of the Congo and further north on Lake Albert in Uganda [36, 38] while *B. sudanica* has only been described from the northern part of Tanzania, including Lake Victoria [6, 36]. *Biomphalaria choanomphala* has been thought to be endemic to Lake Victoria [6] while *B. alexandrina* is native to Egypt so its presence is less likely in Tanzania. A previous study compared relative susceptibility of widespread *B. pfeifferi* and *B. sudanica* with the more range-restricted *B. stanleyi*, *B. smithi* and *B. choanomphala* (which in Uganda are restricted to Lakes Albert, Edward and Victoria, respectively) when challenged with *S. mansoni* genotypes sampled from Lakes Albert and Victoria [39]. This revealed that the survival of infected snails increased when challenged with parasite strains sampled from a different geographical region, suggesting that geographical range expansions of snails could increase risk of schistosome transmission if snails are exposed to new parasite strains with which they have not previously been co-evolving.

The particular communities of parasites and vectors present could thus influence transmission dynamics. While some of the snails at Gombe were confirmed to be *B. pfeifferi*, *B. smithi*-like snails were also present. Since no snails in the park stream sampled shed schistosomes and only three were PCR positive, this could suggest that competition among potential vectors could affect transmission dynamics within a limited stream environment [37]. It is also informative that more than one genotype of *B. pfeifferi* was found among the snails sampled. For *B. glabrata*, which has its main distribution in South America, both resistant and susceptible genotypes of the snail can co-occur within a limited geographical region [4]. So, to increase understanding about the complex relationships among parasites, vectors and hosts [40], it could be important to identify not only the species of snails present but population genetic structure within species of snails that might impact relative susceptibility or vector competence [41].

### Distribution of trematodes based on cercarial shedding and molecular identification

If the present conclusions about prevalence had been based on amplification of the 500 bp band typical of *S. mansoni*, it would appear that the PCR technique was more sensitive than cercarial shedding, with schistosome infection detected in snails at an overall prevalence of 47% compared to only 12%, respectively (Table 1, Fig. 2). This was particularly apparent at Bugamba, where no snails shed larvae but nearly a third of the snails tested showed amplification of the target band and at Mwangongo, where PCR and shedding showed dramatic differences. It is intriguing that both of these villages showed presence of the 1000 bp band and that no samples showing presence of the 1000 bp band shed *S. mansoni* larvae, even though a relatively large proportion also showed amplification of the 500 bp band. The closest similarity of the 1000 bp band was to trematodes with fishes as final hosts [34] but we could not distinguish whether this was a parasite present in the surrounding water or another trematode using snails as an intermediate host. However, this emphasises that relying only on shedding reduces the potential to consider the consequences of mixed infections.

While increased sensitivity and utility of PCR is consistent with many other studies [12, 16, 37, 39, 42–46], almost all have been based only on PCR-based identification, rather than sequencing. In our study, all three 500 bp amplicons sequenced from Kigoma showed amplification of a trematode other than *S. mansoni* (most similar to *Macroderoides spiniferus,* which has been reported to infect snails in Florida, with a fish final host [47]). Since the presence of *B. pfeifferi* was not confirmed through sequencing in this village, it is possible that the *B. smithi*-like snails found there transmit a different species of trematode. Competition among parasite genotypes or species within hosts is well known to affect fitness or virulence [48, 49] and so these findings could have important implications for transmission dynamics of schistosomes. Our results thus emphasise the importance of sequencing to confirm species identification of both intermediate hosts and parasites.

Our finding that the general primers used to simultaneously target both host and parasite also amplify other parasites present suggests that they provide a useful tool for assessing the role of intermediate hosts/vectors in transmitting multiple species of parasites. While other studies have recommended multiplex PCR [45] or qPCR [49], we have demonstrated the ease of using a single PCR reaction for identifying mixed infections. Multiplex PCR (either using specific primers in the same region to target different species or multiple gene regions to increase sensitivity) can be powerful but risk of differential amplification of products, hybridisation between products, and difficulties in resolving amplicons of similar size can create added complications in interpretation [50]. Our approach also has the advantage that amplification of the host DNA can act as an internal control for DNA quality, which is more difficult using multiplex approaches. qPCR can be extremely useful for targeting particular known species but is normally too specific to identify unknown parasites in mixed infections. Using any of these approaches, if it is important to know which species of parasites or vectors are present, sequencing is still critical to confirm identity, particularly when closely related species might have overlapping distributions. In our study, in terms of percent sequence similarity, *S. rodhaini* was the only species that fell within the range of *S. mansoni* isolates, although in terms of absolute differences there were more bp substitutions in the former. This suggests that use of the ITS region alone might not be sufficient to distinguish species within the mansoni group, but should be able to distinguish between other trematodes present. Sequencing of another variable region, such as the mitochondrial gene cytochrome *c* oxidase subunit 1 could help to distinguish closely related species or populations of *S. mansoni* sampled from different geographical regions [51]. Resolving hybrids between trematode species [52] might also not be possible using our method because the ITS region can undergo concerted evolution [53]: our sequences were 100% identical to hybrids between *S. mansoni* and *S. rodhaini*, which could suggest parental dominance in the rDNA repeats. We would thus recommend that the single PCR product approach provides a useful and cost-effective tool for resolving multiple infections but that more targeted approaches would need to be developed for fine-scale species identification within groups, based on extensive sampling across the range of each focal species and detailed analysis of cross-amplification [54].

### Distribution of infected snails in relation to *S. mansoni* prevalence in humans

We found the highest abundance of snails at Mwamgongo, which was the only site where snails infected with *S. mansoni* were found both in the wet and dry seasons, with particularly high prevalence of the 500 bp trematode band in the former (Fig. 3). This is also the village where the highest infection levels with *S. mansoni* were found in humans (68%) in a parallel study, based on morphological identification of the parasites [10]. Schistosome-infected snails were also found in Bugamba, which also showed high prevalence of schistosomiasis in humans (61%). In contrast, the lowest prevalence was found in Mtanga (19%), where no *B. pfeifferi*-like snails were found. These observations are consistent with results from other studies, which have associated the focal distribution of schistosomiasis in humans to localized distribution and infection status of snails [55, 56]. Even with the snapshot sampling that we used, the PCR-based screening of schistosome prevalence in snails could thus provide a useful screening method for predicting relative risk without more invasive human sampling.

## Conclusions

Our study has demonstrated the power of combining PCR-based screening to diagnose parasite infections with sequencing to confirm the particular species of vectors and parasites interacting in a given region. The detection of multiple species of snails that have been implicated as intermediate hosts of *S. mansoni* in samples in a limited geographic region and the presence of multiple trematode species indicate the importance of not relying only on amplification of PCR products of a given size for surveillance informing intervention programmes for schistosomiasis and other infections. It also adds to the evidence that cercarial-shedding should not be relied on as a quantitative measure of relative prevalence, particularly when multiple species of parasites might be present in a region.

## Additional files

Additional file 1: Table S1. Summary of sample sizes of snails collected for experimental shedding (number of snails collected) and PCR, along with the numbers of individuals per site within each sampled village that shed parasites or showed presence of the 500 bp band expected for *S. mansoni* and the 1000 bp band identified as another trematode. Also indicated is the number of PCR samples for which the snail band did not amplify (no. of snails negative for snail band); these were excluded from calculations of percentages for PCR-positive results. The sampling date, the season of sample collection, the stream name, and location of the villages are also indicated. (XLSX 15 kb)

Additional file 2: Table S2. Sampling locations and infection status of snails found in Gombe National Park and surrounding villages, showing the village/site and sampling points along the focal stream within each village, the individual code for the sample, the season sampled and whether schistosomes were detected through experimental shedding (microscopy) or presence of the 500 bp PCR band. Presence of the 1000 bp band is also indicated, along with the species identification of snails and parasites based on sequencing. Mtanga stream in Mtanga villages where no snails were found has been excluded from this list. (XLSX 24 kb)

Additional file 3: Supplementary Sequence Information. This file provides the sequences (in FASTA format) that were obtained from sequencing each of the band sizes found using the primers ETTS2 and ETTS17, which target the ITS1 region of the ribosomal DNA array. For the 1000 bp band, the sequences were not of sufficient quality to sequence through the entire fragment. For the other bands (approximately 600 bp for snails and 500 bp for trematodes), the size of the consensus sequence for the two primers is indicated, along with the sequence identity, as determined using BLAST. For the 1000 bp product, only the sequence from the ETTS2 primer is provided. For *B. pfeifferi*, the type 1 and type 2 sequences only differ by a single nucleotide. For each sequence type, the closest matching sequences in GenBank are included. Alignment is only possible within and not between, sequence types, due to the large length variation. (TXT 20 kb)

## Abbreviations

BLAST: Basic Local Alignment Search Tool
ITS: internal transcribed spacer
NCBI: National Centre for Biotechnology Information
NIMR: National Institute for Medical Research
